# Exploring the Link Between Cognitive Abilities and Speech Recognition in the Elderly Under Different Listening Conditions

**DOI:** 10.3389/fpsyg.2018.00678

**Published:** 2018-05-11

**Authors:** Theresa Nuesse, Rike Steenken, Tobias Neher, Inga Holube

**Affiliations:** ^1^Institute of Hearing Technology and Audiology, Jade University of Applied Sciences, Oldenburg, Germany; ^2^Cluster of Excellence “Hearing4All”, Oldenburg, Germany; ^3^Medizinische Physik, Oldenburg University, Oldenburg, Germany; ^4^Faculty of Health Sciences, Institute of Clinical Research, University of Southern Denmark, Odense, Denmark

**Keywords:** speech recognition, cognition, complex listening conditions, working memory, attention, hearing loss

## Abstract

Elderly listeners are known to differ considerably in their ability to understand speech in noise. Several studies have addressed the underlying factors that contribute to these differences. These factors include audibility, and age-related changes in supra-threshold auditory processing abilities, and it has been suggested that differences in cognitive abilities may also be important. The objective of this study was to investigate associations between performance in cognitive tasks and speech recognition under different listening conditions in older adults with either age appropriate hearing or hearing-impairment. To that end, speech recognition threshold (SRT) measurements were performed under several masking conditions that varied along the perceptual dimensions of dip listening, spatial separation, and informational masking. In addition, a neuropsychological test battery was administered, which included measures of verbal working and short-term memory, executive functioning, selective and divided attention, and lexical and semantic abilities. Age-matched groups of older adults with either age-appropriate hearing (ENH, *n* = 20) or aided hearing impairment (EHI, *n* = 21) participated. In repeated linear regression analyses, composite scores of cognitive test outcomes (evaluated using PCA) were included to predict SRTs. These associations were different for the two groups. When hearing thresholds were controlled for, composed cognitive factors were significantly associated with the SRTs for the ENH listeners. Whereas better lexical and semantic abilities were associated with lower (better) SRTs in this group, there was a negative association between attentional abilities and speech recognition in the presence of spatially separated speech-like maskers. For the EHI group, the pure-tone thresholds (averaged across 0.5, 1, 2, and 4 kHz) were significantly associated with the SRTs, despite the fact that all signals were amplified and therefore in principle audible.

## Introduction

Under real-life listening conditions, background noise is typically present and hinders effective communication, especially if any of the dialogue partners is suffering from a hearing loss. During diagnostics and rehabilitation of hearing impairment, tests of speech recognition in quiet and in noise (e.g., Kollmeier and Wesselkamp, 1997; Wagener et al., 1999) are performed to determine the degree of hearing loss and to verify the benefit of hearing devices. It is well-known that the presence of interfering noise (e.g., Hällgren et al., 2005), as well as peripheral auditory deficits, adversely affect speech recognition performance (e.g., Bronkhorst and Plomp, 1992; Humes, 2013). Furthermore, there is evidence that performance on speech recognition tasks also depends on variations in cognitive abilities (Hunter and Pisoni, 2018). Also, correlational studies indicate that outcomes of speech-in-noise recognition tasks are related to cognition (CHABA, 1988; Akeroyd, 2008; Besser et al., 2012). To examine which particular cognitive functions are most related to speech recognition and therefore should be included in the present study, the recent literature of correlational studies investigating the link between both were reviewed. In particular, working memory has been shown to be related to speech recognition in noise in groups of young normal-hearing adults (e.g., Zekveld et al., 2013, mean age: 23 years). For elderly participants with different hearing status the findings are mixed, indicating a large influence of the age and hearing loss group studied (see Akeroyd, 2008; Füllgrabe and Rosen, 2016b for an overview). Because of the age-dependency of hearing loss, controlling age is necessary in investigations concerning the interrelationship. Other studies have identified an influence of attentional abilities on speech-in-noise recognition in young normal-hearing (Oberfeld and Klöckner-Nowotny, 2016, age range: 18–30 years) and in elderly participants. In the elderly, participants with a wide range of hearing thresholds (Cahana-Amitay et al., 2016, age-range: 55–84 years) as well as groups with mild hearing loss without aiding (Heinrich et al., 2015, age range: 50–74 years) or mild-to-moderate hearing-impaired hearing aid wearers were examined (Heinrich et al., 2016, age range: 50–74 years). The results indicate that the relationship between attention and speech recognition is to this extent independent of age and hearing loss. Moreover, semantic knowledge and the vocabulary of young, normal-hearing listeners (Kaandorp et al., 2016, mean age of groups: 24–29 years; Carroll et al., 2015b, age range: 18–34 years) was recently examined in this context (see Besser et al., 2013 for an overview). Executive functioning, especially inhibitory control, may be an additional factor contributing to speech recognition, as indicated by Ellis et al. (2016) using a large sample of ~1,500 participants (age range: 18–91 years, mean age: 63 years).

Some cognitive abilities change with age. In speech recognition measurements, age and cognition were observed to be interacting factors (e.g., Füllgrabe et al., 2015; Gordon-Salant and Cole, 2016). For example, Gordon-Salant and Cole (2016) found a negative effect of age between a younger (18–25 years) and an older (61–75 years) subgroup of normal-hearing participants that had a small working memory capacity but not for participants with large working memory capacity. In addition, some studies indicated that untreated audiometric hearing loss can reduce cognitive abilities. For example, Lin et al. (2011, 2014) found that brain volume and cognitive functioning were associated with the degree of audiometric hearing loss. This suggests that hearing loss can impact speech recognition not only via peripheral auditory deficits but also via reduced cognitive abilities (e.g., Desjardins and Doherty, 2013; Smith and Pichora-Fuller, 2015; Meister et al., 2016). Even if audiometric hearing loss is compensated for through the provision of hearing aids, the effects of cognition on speech recognition can be overshadowed by the effects of audiometric hearing loss (Heinrich et al., 2016). Furthermore, hearing aids by themselves and/or the acclimatization to amplification might have an impact on cognition. Habicht et al. (2016) found that inexperienced hearing-aid users differed in terms of cognitive-linguistic speech processing abilities from experienced users. Furthermore, in a subsequent longitudinal study these authors found that the provision of hearing aids to inexperienced users substantially improved their speech processing abilities after 24 weeks of hearing aid use (Habicht et al., 2017). These results indicate that long-term amplification may lead to restored cognitive abilities in hearing-impaired persons.

Hearing loss is generally described by pure-tone thresholds, but in addition more central processes of hearing are also involved. There is evidence that supra-threshold auditory processing deficits in abilities such as sensitivity to temporal-envelope and temporal-fine-structure information decline with age, and this happens even in the absence of audiometric hearing loss (e.g., Füllgrabe, 2013; Füllgrabe et al., 2015). This might partially explain the speech-in-noise perception deficits observed for older, normal-hearing listeners (Füllgrabe et al., 2015).

Apart from that, degradation of speech (due to hearing loss or masking) is thought to cause a higher cognitive load. This is incorporated in the ease-of-language-understanding model (ELU), which assumes that clearly audible, undistorted signals can be perceived and processed very quickly, while degraded signals lead to an activation of higher cognitive abilities (Rönnberg et al., 2013). In experimental EEG studies, evidence for higher cognitive load (represented by alpha power enhancement) in listening to degraded signals was found for young, normal-hearing listeners (20–32 years, Obleser et al., 2012) as well as older listeners (62–86 years) with and without hearing loss (Petersen et al., 2015). A recent meta-analysis that focused on the relationship between speech recognition and working memory as measured with a reading span test, indicated that this assumption might not hold for every group of participants (Füllgrabe and Rosen, 2016b). Because these authors did not consistently find a link between verbal working memory and speech recognition in noise, they recommended providing information about the age and hearing loss of an analyzed sample.

In recent studies the properties of the masker signals have also been found to influence the relationship between the cognitive performance and speech recognition. For example, measurements of speech recognition in quiet seem to result in smaller correlation coefficients than measurements in noise for young normal-hearing participants (20–33 years, Moradi et al., 2014). Competing speakers seem to result in a closer link of cognition and speech recognition than stationary noise maskers in different samples (Besser et al., 2013; Heinrich et al., 2016). Two different mechanisms of masking with competing speakers compared to stationary noise may lead to informational masking and dip listening caused by modulations of the masker signal. Informational masking is defined here as the additional amount of masking due to semantic information introduced into a scene. This type of masking is frequently considered to address more central structures in contrast to energetic masking that is often equated with peripheral masking (Durlach et al., 2003). Dip listening is the opportunity to perceive glimpses of the target speech in short silent intervals of the masker signal. In single-speaker or speech-like maskers the opportunity to listen in the dips leads to better SRTs in speech recognition tests (Festen and Plomp, 1990; Holube et al., 2011). Furthermore the complexity of the target speech signal might influence the relationship between cognition and speech recognition. Heinrich et al. (2015) showed for elderly participants that had mild hearing loss without hearing aids that the use of linguistically more complex speech material in terms of digits and sentences led to a stronger relationship between cognitive abilities and speech-in-noise performance compared to a phoneme-discrimination task. In contrast, other studies showed that the link between cognitive factors (especially working memory) and speech recognition might not be affected by the linguistic complexity for normal-hearing listeners of different age groups (Füllgrabe et al., 2015; Füllgrabe and Rosen, 2016b). Some findings indicate that the interaction between age and the cognitive abilities describing the putative link to speech recognition are moderated by the linguistic complexity of the speech signal (Gordon-Salant and Cole, 2016). Neher et al. (2009) examined the role of working memory and attention for spatially-separated competing speech signals or stationary speech-shaped noise with a group of hearing-impaired subjects that used hearing aids (28–84 years, mean age: 60 years). They found a stronger relationship between cognitive abilities in the complex listening task compared to a SRT measurement with a stationary, co-located noise as masker signal. In a multiple regression analysis, they also found cognition to be more predictive than audiometric outcomes in a front-back masker condition. Overall, it can thus be hypothesized that listening conditions characterized by high complexity due to the use of a linguistically complex speech material or competing speech signals lead to a closer link between cognitive abilities and speech recognition.

The current study aimed to address the link between speech recognition in noise and cognitive abilities in different listening conditions. Age-matched groups of older adults with either age-appropriate hearing or hearing impairment were examined to explore the relationship between cognitive abilities and speech recognition using complex masker signals and a broad test battery of cognitive testing. The listening conditions were designed to study how the effects of cognition on speech recognition performance change by introducing “dip listening” (Festen and Plomp, 1990), spatial separation among the target speech and masker signals, and “informational masking” (Durlach et al., 2003; Koelewijn et al., 2014). Different types of masker signals can be categorized in terms of energetic, modulation and informational masking (Stone et al., 2011, 2012). In this study, all maskers contained a certain amount of modulation, including short pauses and thus the opportunity of dip listening. Therefore purely energetic maskers (e.g., stationary noise) were not included. Informational masking as defined here corresponded to the introduction of semantic information into the masker signals rather than the introduction of the auditory object segregation that is available in several of the masking conditions. In addition to the different masker conditions, this study also included a neuropsychological assessment. This assessment was performed with the goal of investigating four types of cognitive abilities that are expected to influence speech recognition under the different masking conditions: (1) verbal working and short-term memory, (2) selective and divided attention, (3) executive functioning, and (4) lexical and semantic abilities. As described above, in a number of studies working memory was found to be related to speech recognition in noise in older adults with hearing impairment, leading to the inclusion of three working memory tests in the neuropsychological test battery. The attention and executive functioning tests were included to obtain further information regarding how the allocation of spare cognitive capacity (Kahneman, 1973), being the remaining cognitive capacity when performing an effortful task, is connected to performance with spatially-separated signals. Furthermore, given that it was hypothesized that informational masking would lead to a stronger link of lexical and semantic abilities to speech recognition, the latter were also included in the assessment. To contrast the effects of hearing loss on the link between speech recognition and cognitive abilities, older adults with either age-appropriate hearing or hearing impairment were included in this study. The two groups were matched both in age and gender. In order to restore, at least partially, audibility of the target and masker signals, hearing aids were provided to all hearing-impaired participants. Using correlation and regression analyses, the links between cognitive abilities and speech recognition in the different listening conditions and any contribution of hearing loss to this interaction were explored. In view of the literature findings summarized above, a stronger link between cognitive abilities and speech recognition was expected for the more complex listening tasks, particularly so for the hearing-impaired group due to the degraded speech information provided by the hearing aids and supra-threshold processing along the auditory pathway. Overall, measurements were conducted addressing two research questions:

1. Which cognitive abilities link speech recognition in complex listening conditions of the elderly participants?
2. How does this link vary if hearing impairment is present?

## Materials and methods

### Participants

**Figure 1:**
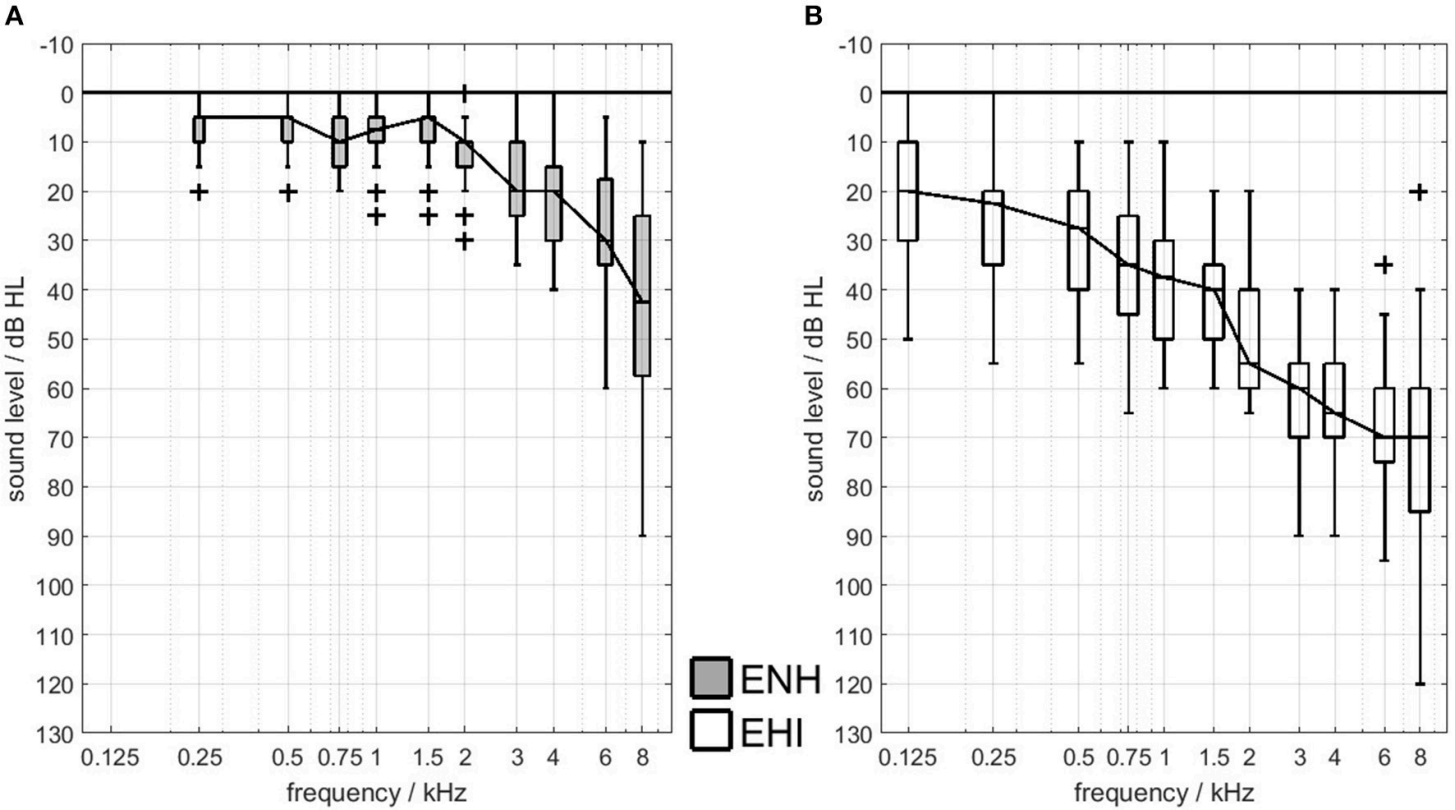
Better-ear hearing thresholds for the ENH group **(A)** and the EHI group **(B)**. Lines connect medians and boxes indicate the 25th and 75th percentiles. “+” indicates outliers.

For the current study, a total of 46 elderly participants were recruited from the voluntary test subject database of the Hörzentrum Oldenburg GmbH. Inclusion criteria were German as the native language and a visual acuity of at least 0.63, since good visual acuity was crucial for some of the neuropsychological testing. The participants' (corrected) near-field vision was tested with Landolt rings (Optovist V20.009, Vistec AG, Olching, Germany), resulting in the exclusion of five participants from the study. The 41 remaining participants were aged from 60 to 77 years and were divided into two groups on the basis of their audiometric hearing thresholds, namely older adults with either age appropriate hearing (ENH, *N* = 20) or hearing impairment (EHI, *N* = 21). The groups were nearly matched in age and gender (no significant age differences between groups, Mann–Whitney-*U*-Test, p = 0.6). The ENH group was aged 60–75 years (mean: 67.65 years, *SD*: 4.8, 13 females) and the EHI group was aged 61–77 (mean: 68.76, *SD*: 5.9, 9 females). The better-ear hearing thresholds averaged across 0.5, 1, 2, and 4 kHz (PTA4) of the ENH group were on average 10.6 dB HL (*SD*: 3.9 dB HL, min: 1.5 dB HL, max: 17.5 dB HL). The audiometric cut-off for inclusion in the ENH group was set to a PTA4 of max. 25 dB HL in both ears and no need for a hearing aid provision according to the German guidelines (G-BA, 2017). All participants in the EHI group had mild-to-moderate, symmetrical sensorineural hearing losses (mean PTA4: 42.4 dB HL, *SD*: 8.4 dB HL, min: 25.0 dB HL, max: 53.75 dB HL) and at least one year of hearing-aid experience (mean: 6.9 years, *SD*: 5.0 years). Audiometric symmetry was characterized by across-ear differences of max. 15 dB at every standard audiometric frequency between 125 and 8,000 Hz. The air-bone gap was max. 15 dB at 0.5, 1, 2, 4, and 6 kHz. Air-conduction pure-tone hearing thresholds of the participants' better ears are shown in Figure 1.

During the speech recognition tasks, all EHI listeners wore identical receiver-in-the-canal hearing aids with double domes (Siemens Pure micon 7mi, M-Receiver) that are similar to the devices with which they were fitted in daily life. The hearing aids were in the omnidirectional microphone mode and were fitted bilaterally according to the NAL-NL2 formula (Keidser et al., 2011). According to the data sheet of the manufacturer, the attack and release times of the 20-channel dynamic range compressor were 3 and 90 ms. All comfort settings were deactivated and feedback cancellation was only activated if necessary. The participants were free to choose which hearing aids were worn during anamnesis and cognitive testing (their own or the ones offered). None of the participants had clinically relevant deficits in the cognitive areas, as monitored by a neuropsychologist. An hourly rate was paid and all participants gave their informed consent prior to inclusion in the study. The experiment was approved by the ethics committee (“Kommission für Forschungsfolgenabschätzung und Ethik”) of the Carl von Ossietzky University in Oldenburg, Germany (Drs. 22/2014).

### Test protocol

Measurements were conducted during three visits of ~2 h duration each and with at least 2 days between two consecutive visits. During the first visit, overall anamnesis, otoscopy, pure-tone audiometry, and visual accuracy test were administered. During the second visit, the speech recognition measurements for the different conditions were performed in randomized order. The attentional tests were also administered in randomized order. During the third visit, all the other cognitive tests were completed in randomized order. The pure-tone audiometry was carried out in a sound-attenuating booth. The other measurements took place in two different, quiet rooms.

### Speech recognition in noise

**Figure 2:**
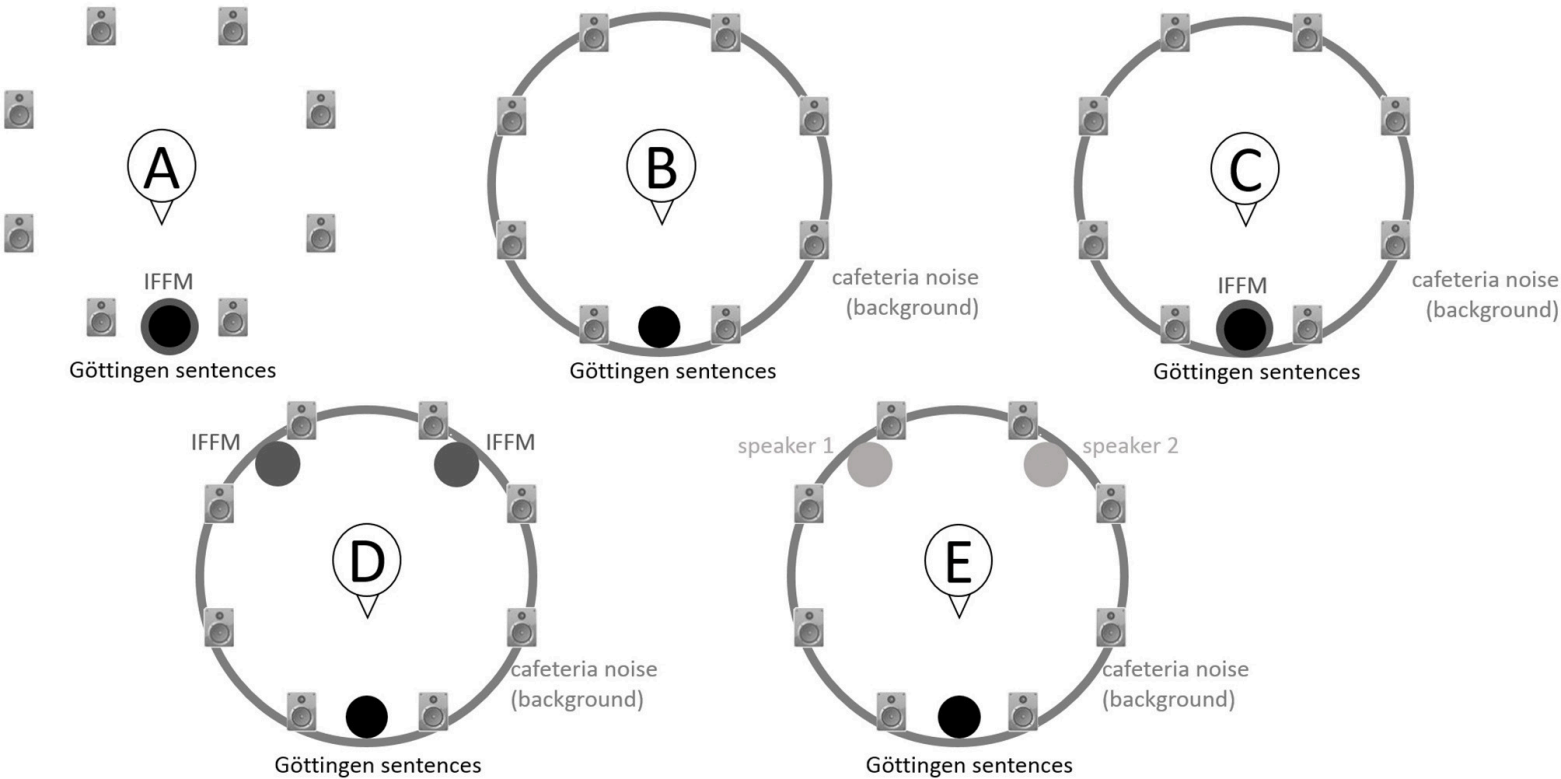
Schematic overview of the five listening conditions for which SRT measurements were conducted to examine speech recognition in a realistic cafeteria situation **(B)** and the influence of listening in the dips (**A** vs. **C**), spatial separation (**C** vs. **D**), and informational masking (**D** vs. **E**).

Using the adaptive procedure of Brand and Kollmeier (2002) for measuring the SRT corresponding to 50%-correct speech recognition in noise, a German sentence test with everyday-life sentences (Göttingen Sentence Test, e.g., “Engines need petrol, oil and water,” Kollmeier and Wesselkamp, 1997) was performed. Each test list consisted of 20 sentences. One list per test condition or SRT measurement was carried out. Participants were asked to repeat the sentences to the examiner, but no feedback was given during the test. The starting SNR was 0 dB. The target signal was a sentence uttered by a male speaker presented from in front (0° azimuth) at 65 dB SPL. The SRT measurements took place in a free-field setup consisting of eight KRK Systems Rokit 5 loudspeakers (Deerfield Beach, USA) positioned at 1.5 m distance and at angles of 45° relative to each other, starting with an azimuthal angle of 22.5° relative to the listening position (see Figure 2). A higher-order Ambisonics-based software toolbox (Grimm et al., 2015) was used for simulating five different listening conditions. The different masker signals were continuously presented during each SRT measurement. The first sentence started 5 s after the onset of the masker signals. In condition A, the target speech and a point-source masker (International Female Fluctuating Masker, IFFM; Holube, 2011), a variation of the International Speech Test Signal (ISTS; Holube et al., 2011) were presented from 0° azimuth. The IFFM masker consists of speech snippets in six different languages (all female speakers) and is an unintelligible speech masker that preserves the spectrum and temporal characteristics of real speech. In condition B, spatially-diffuse cafeteria noise was used as the masker, to generate a more complex and realistic listening condition. In conditions C to E, different point-source maskers were presented together with the diffuse cafeteria noise. Condition C combined the maskers of conditions A and B, thereby filling the temporal dips of the IFFM with cafeteria background noise and introducing greater spatial complexity into the listening condition. By comparing conditions A and C, the participants' ability to “listen in the dips” was assessed. In condition D, the IFFM masker was spatially separated from the target speech by presenting the masker alternatingly at −135° and +135° azimuth with a change in position every 1.5–4 s (mean: 3 s). In condition E, more informational masking was introduced by presenting a realistic conversation of two female German speakers. More specifically, the two speakers performed a Diapix task (Van Engen et al., 2010), in which they solved a spot-the-difference puzzle. The conversation was chosen out of 14 recordings with the following characteristics: two female speakers, as balanced contributions of both speakers as possible, as few filler words and additional noise (e.g., laughing, breathing) as possible. Additionally, the recordings were processed such that there were no long overlaps between the two speakers.

For all listening conditions, the target and masker signals were calibrated individually to 80 dB SPL unweighted at the listener's position and attenuated internally to the presentation level of 65 dB SPL (speech signal). Calibration signals were either a speech-shaped noise provided by the authors of the Göttingen Sentence Test (Kollmeier and Wesselkamp, 1997; target, cafeteria masker) or the IFnoise, which has the same long-term spectrum as the ISTS (Holube et al., 2011; IFFM, conversation).

### Neuropsychological test battery

The neuropsychological test battery included tests for verbal working and short-term memory (MEM), selective and divided attention (ATT), and executive functioning (EX), as well as lexical and semantic abilities (LEX). In cognitive test theory, the different generic cognitive functions are constructs that could be approached by various measurement tools. These result in measurable changes in the dependent variables such as reaction times or number of mistakes (Wirtz, 2014). To substantiate the findings with regard to the underlying cognitive functions, at least two tests addressing the particular function were performed for each of the four types of cognitive ability. All cognitive tests were chosen to use either visual stimuli or simple auditory stimuli that could, empirically observed, be perceived effortlessly by aided hearing-impaired persons.

#### Working memory

##### Digit span

The digit span task from the working memory subset of the Wechsler Adult Intelligence Scale—Fourth Edition (WAIS-IV; Petermann, 2012) was used. In this test, the examiner read short sequences of digits with a speed of approximately one digit per second. The participant's task was to repeat sequences of numbers either in the same order (forward), in reverse order (backward), or in ascending (sorted) order. The difficulty was increased as the length of the series of numbers increased after two repetitions, and the test was stopped when both sequences of numbers of the same length could not be repeated correctly. The longest sequence contained eight (backward) or nine (forward and sequential) digits. The outcome variables were the span scores (total number of correctly repeated sequences) for each of the three conditions tested, with a maximum score of 16 each.

##### Reading span

To examine verbal working memory capacity, a German version of the reading span test (Carroll et al., 2015a) was used. The test material contained 54 short German sentences with simple sentence structures (e.g., “The farmer picks the apples.”) that were either plausible or absurd. The sentences were presented on a computer screen one segment at a time every 800 ms. Participants had to read aloud each sentence, decide whether or not it was plausible (within max. 1,750 ms) and repeat the first or final segment of each sentence after the presentation of three to six sentences. The information about which part of the sentence (first or final) had to be repeated was only given to the participants after the presentation of all three to six sentences. The outcome variable was the percentage of correctly repeated words (in the correct order), as recommended by the test developers.

##### 2-Back task

The 2-back-task from the test battery for attention measures (TAP, Zimmermann and Fimm, 2013b) was used. It contained the digits 1–9, which were shown on a screen for 3 s each. The task of the subjects was to tap a switch if the current number was the same as the one given two presentations earlier. The outcome variables were reaction time (RT) as well as the number of mistakes that were made during the examination.

#### Attention

##### WAF-S

For assessing selective attention, a test from the Perception and Attention Battery (Häusler and Sturm, 2009) of the Vienna Test System (Wiener Testsystem, SCHUHFRIED GmbH, Austria) for neuropsychological assessment was performed. In this test, relevant stimuli (circles and squares) and irrelevant stimuli (triangles) were presented on a screen for 1,500 ms. The participant had to react with a keystroke to changes in relevant stimuli (lighter or darker) that appeared 500 ms after the initial presentation. The RTs were measured by a module in the response panel, thereby avoiding the latencies resulting from the performance of the computer. Additionally to the RTs, the total number of errors was reported.

##### Ruff 2&7

The Ruff 2&7 selective attention test was developed by Ruff and Allen (1996) and is a pen-and-paper test. The task of the participant was to cross out any 2 and 7 s in an array of other letters and digits. On the test sheet, 20 pseudo-random sections (10 containing digits, 10 containing letters) were printed. The participant had 15 s to complete each section. The outcome variables were the speed and accuracy of each participant's performance, which was calculated by summing up the number of correctly deleted digits and dividing it by the total number of mistakes.

##### TAP—divided attention test

For assessing divided attention, a cross-modal procedure from the test battery for attention measures (TAP, Zimmermann and Fimm, 2013b) was used. In this test, two tasks have to be carried out in parallel. The tasks were divided into two synchronously presented stimulus channels (visual and auditory). The participant had to react to both target stimuli by pressing a key. The visual task was presented on a screen and included the monitoring of a square arrangement with a dot pattern at 16 possible positions. Every 2 s, six to eight crosses appeared, forming different patterns. If the crosses were arranged at four contiguous positions so that they form a square, the participant had to press the response button. For the auditory task, two sinusoidal tones (450 and 1,070 Hz) were alternately presented at a rate of 1 s through loudspeakers. The task of the participant was to press the response button when the given tone was presented twice in a row. The outcome variables were the auditory and visual RTs, as well as the number of errors.

#### Executive functioning

##### Trail making test (TMT)

The Trail Making Test introduced by Reitan (1992) was used to investigate the participants' executive functioning. The pen-and-paper test for adults consisted of two parts (A and B), with 25 circles containing numbers or letters. The aim was to connect the circles as fast as possible. In test part A (TMT-A), the numbers from one to 25 were to be linked in ascending order. Since the task in this subtest was relatively easy, especially motor skills and the processing speed were assessed. In the second part (TMT-B), the numbers from one to 15 and the letters A to L were to be connected alternately in ascending order (1-A-2-B-3-C etc.). This task required primarily cognitive flexibility and executive functions. The outcome variable was the time spent on each test section.

##### STROOP

The STROOP test was performed with an implementation from the Vienna testing system (SCHUHFRIED GmbH, Austria), consisting of four test parts (Puhr and Wagner, 2012). The task of the participants was to either read or name the right color of a word or bar presented on a screen and to press the appropriate color button. In the first two parts, the processing speeds of reading the color names (printed in gray) and of naming the colors (colored bars) were measured. In the interfering conditions (part 3 and 4) the words “yellow,” “green,” “red,” and “blue” were presented on the screen but printed in a different color. In the third part, the participants were asked to read the color word and press the right button whereas in the fourth part, the color of the word was to be named. With respect to the baseline speed, the reading and naming interference were calculated by subtraction.

#### Lexical and semantic abilities

##### Lexical decision task (LDT)

In the Lexical Decision Test (LDT), the lexical processing time was investigated, which required matching simple words with the lexical memory and classify them based on the categories “plausible” and “absurd” (Carroll et al., 2015b). Four letters that formed either an existing German word (e.g., “Raum”) or a phonologically plausible but invented non-sense word (e.g., “Lauk”) were shown on a screen to the participants. The participants had to press a button, indicating whether the word exists in German or not. Simple color-coded (blue for “right,” red for “wrong”) USB switches were used in this test. The color arrangement was also used for the response boxes on the screen, and each was enhanced shortly after the respective button has been pressed. The subjects were told to respond as quickly as possible, although no time limit was implemented in the procedure. The speech material consisted of 40 non-words, 20 frequently occurring German words and 20 rare German words. The difference between the time elapsing in the recognition of non-words during lexical analysis (without success) and the (processing) time for a successful access when the word was correctly recognized, was reported as the RT difference of the logarithmic RTs.

##### Text reception threshold (TRT)

The measurement of the TRT was mainly performed as described by Zekveld et al. (2007). In this test, the threshold for a reading comprehension of 50% was measured as a function of the percentage of the text area obscured. In the present study, an implementation of the TRT-test was used, which had been developed in the Department of Medical Physics (University of Oldenburg, Germany). The speech material was derived from three test lists of the Göttingen sentence test (Kollmeier and Wesselkamp, 1997) that had been excluded from the SRT measurements to avoid repetitions of the test material. Only five-word sentences were combined to a set of twenty sentences consisting of 25–29 characters each. The sentences appeared word by word and in red color against a white background, while the masker contained black vertical bars. The threshold was determined adaptively with word scoring and an initial coverage of 50%.

##### Vocabulary test (MWT-B)

The vocabulary of the participants was tested using a German multiple choice vocabulary test (MWT-B, Ger.: Mehrfachwahl-Wortschatz-Intelligenztest Teil B) which was originally used as a tool to examine the general level of intelligence (Lehrl, 2005). The pen-and-paper test was carried out on a test sheet containing 37 rows of four imaginary non-sense words and one existing German word that was the target and should be crossed out by the participants. The number of correctly selected words was reported.

## Results

### Speech recognition

**Figure 3:**
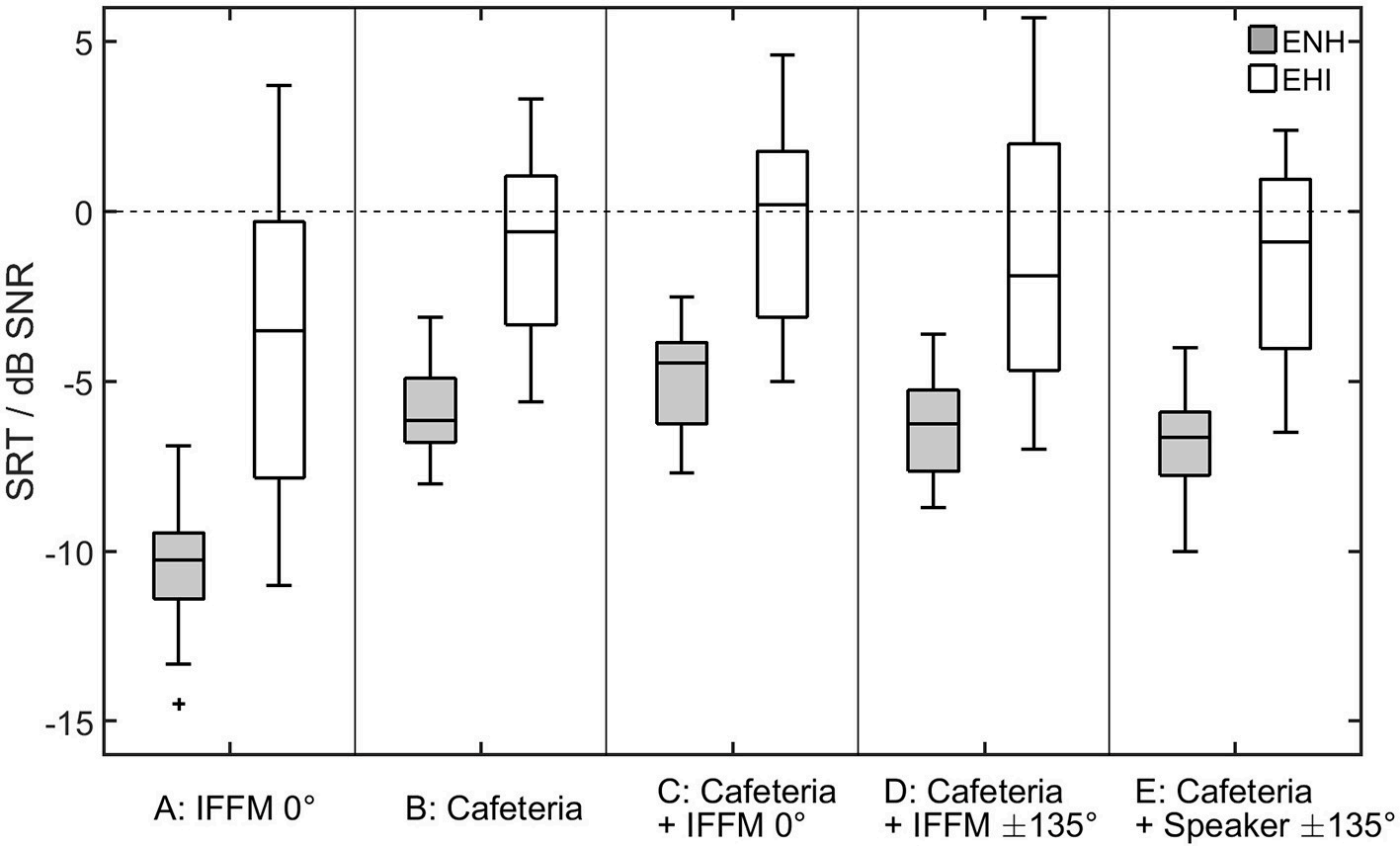
SRTs obtained by the ENH and the EHI group for the five listening conditions (A–E) shown in Figure 2. The horizontal line in each box indicates the median, and the boxes indicate the 25th and 75th percentiles, respectively.

The results of the speech recognition measurements are shown in Figure 3. As the data are not normally distributed (Shapiro–Wilk Test, *p* < 0.016 for all variable), non-parametric statistics were applied for the analyses. SRTs differed, depending on the listening condition [Friedman-Test, $\chi_{\left(4\right)}^{2}$ = 88.1, *p* < 0.001]. To follow up these findings, Wilcoxon tests were used and the Bonferroni-corrected significance level α = 0.005 was applied. The outcomes of the paired comparisons are shown in Table 1. In condition A (IFFM masker only), when listening in the dips was possible, all participants showed lower (better) SRTs compared to each of the other conditions (see Table 1). The added spatial separation in condition D also led to significantly better SRTs compared to condition C. Nevertheless, no significant difference was found for the comparison of the speech-like masker in condition D and the real conversation in condition E.

**Table 1:** Results of the *post-hoc* paired comparisons (Wilcoxon-Test) for all listeners and each condition (rows 2–5) and results of the group comparison (Mann–Whitney-*U*-Test) exploring the differences between NH and HI groups for each listening condition; significant effects are labeled with an asterisk (^*^) as referred to the Bonferroni-corrected significance levels.

	**Condition A**	**Condition B**	**Condition C**	**Condition D**	**Condition E**
	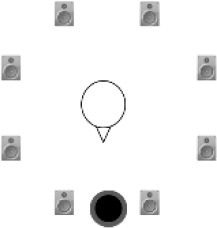	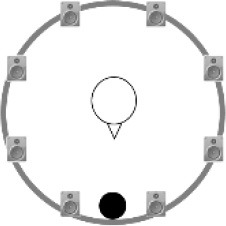	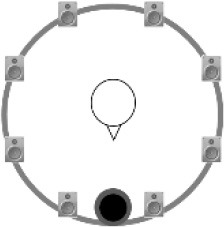	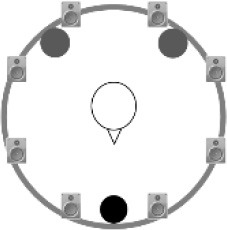	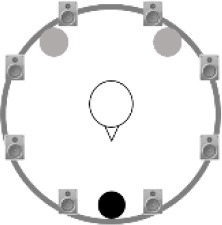
Condition A	NA	T = −5.34, *p* < 0.0001^*^	*T* = −5.52, *p* < 0.0001^*^	*T* = −5.48, *p* < 0.0001^*^	*T* = −5.02, *p* < 0.0001^*^
Condition B	NA	NA	*T* = −3.40, *p* = 0.001^*^	*T* = −0.98, *p* = 0.328	*T* = −3.52, *p* = 0.0004^*^
Condition C	NA	NA	NA	*T* = −3.76, *p* = 0.0002^*^	*T* = −5.05, *p* < 0.0001^*^
Condition D	NA	NA	NA	NA	*T* = −0.53, *p* = 0.011
NH vs. HI	*U* = 37.5, *z* = −4.50, *p* < 0.0001^*^, *r* = −0.70	*U* = 21.0, *z* = −4.93, *p* < 0.0001^*^, *r* = −0.77	*U* = 48.0, *z* = −4.23, *p* < 0.0001^*^, *r* = −0.66	*U* = 43.5, *z* = −4.34, *p* < 0.0001^*^, *r* = −0.68	*U* = 25.5, *z* = −4.81, *p* < 0.0001^*^, *r* = −0.75

There was also a significant group effect. In each listening condition, the SRTs were significantly higher (poorer) for the EHI listeners than for the ENH listeners (Mann–Whitney-*U*-Test, Bonferroni-corrected α = 0.01; see Table 1), despite the fact that audibility was generally ensured by means of the hearing aids.

### Cognitive abilities

The cognitive outcome variables were matched individually to standards (if available). All of the participants had normal, age-appropriate cognitive abilities according to the norm samples (Ruff and Allen, 1996; Tombaugh, 2004; Lehrl, 2005; Petermann, 2012; Puhr and Wagner, 2012; Sturm, 2012; Zimmermann and Fimm, 2013a). Thus, the individual's data points lay within a corridor of ± *1 SD* around the mean of normative data for their age group. For some tests, namely RST, TRT, and LDT, no normative data were available. Table 2 shows the mean results and standard deviations of the neuropsychological data for the two subject groups. Although, the mean values of most cognitive tests suggest that ENH showed a better performance than EHI, a series of paired comparisons revealed no differences between the groups at Bonferroni-corrected significance level (Mann–Whitney-*U*-Test, α = 0.0025). Since within-group standard deviations are relatively high for both groups, most of the paired comparisons did not support significant differences between the two groups, even without adjustment for multiple comparisons.

**Table 2:** Descriptive data of the 20 neuropsychological outcome variables for the ENH and EHI groups.

		**Digit span forward**	**Digit span backward**	**Digit span sequential**	**2-back task (reaction time, ms)**	**2-back task (errors)**	**Reading span (% correct)**	**Ruff 2&7 (speed)**	**Ruff 2&7 (accuracy)**	**TAP (auditive reaction time, ms)**	**TAP (visual reaction time, ms)**	**TAP (errors)**	**WAF-S (reaction time, ms)**	**WAF-S (errors)**	**TMT-A (duration, s)**	**TMT-B (duration, s)**	**STROOP (reading interference, ms)**	**STROOP (naming interference, ms)**	**TRT**	**Vocabulary test**	**LDT (reaction time difference, ms)**
ENH	Mean	9.75	9.30	9.95	652.65	4.84	30.28	263.45	17.30	649.20	835.50	1.35	365.80	2.85	30.95	78.84	0.19	0.28	56.49	31.95	0.28
	*SD*	1.92	2.18	1.67	142.49	2.79	6.62	43.37	13.88	122.19	112.44	1.18	53.51	5.14	7.17	21.70	0.30	0.58	4.87	3.39	0.26
EHI	Mean	8.90	8.71	9.48	746.87	4.95	30.07	229.95	13.43	675.98	899.25	1.24	440.95	3.38	35.42	88.58	0.12	0.21	53.99	32.24	0.24
	*SD*	1.67	1.31	2.09	229.67	3.60	7.26	33.50	11.01	116.86	101.99	1.26	147.06	8.05	13.53	33.20	0.093	0.23	3.22	2.66	0.18

Prior to performing correlation and regression analyses, the number of cognitive outcome variables was reduced by calculating composite scores. Thus, the imbalance between the number of test variables and the number of participants was optimized, resulting in an increase in the statistical power in subsequent analyses. Furthermore, the various interactions between different cognitive outcome variables were separated by calculation of factor values based on at least two different test procedures addressing the same cognitive function. In a first step, each variable was z-transformed and, if necessary, sign inverted to match directions (the higher the score, the better the performance). In a second step, a confirmatory principal component analysis (PCA) of the test variables on the basis of neuropsychological test theory was used. Factor loadings were calculated (see Table 3) for four predefined factors: (1) working and short-term memory (MEM), (2) selective and divided attention (ATT), (3) executive functioning (EX), and (4) lexical and semantic abilities (LEX). For every generic cognitive function measured here, at least two neuropsychological tests were performed. Each factor is meant to represent the shared variance of the tests, which measure a specific cognitive ability, while excluding information about the general cognitive status. To account for a possible speed-accuracy-tradeoff in some testing procedures, two variables of the same test measuring speed (processing time, reaction time) and accuracy (errors) were included in the same factor. Concerning factor 1, loadings of the three components of the digit span test and the error rate of the 2-back task were highest. Rather unexpectedly, the reading span score contributed least to this factor. Concerning the attention-related factor, reaction-time variables showed higher factor loadings, which was also the case with respect to factor 3, for which the outcomes of the STROOP test contributed more than the TMT variables. Concerning factor 4, outcome variables of all three measures showed high factor loadings and therefore contributed to this factor in a relatively balanced manner.

**Table 3:** Factor loadings for each variable included in the confirmatory PCA.

**Factor 1: Working and short-term memory**		**Factor 2: Selective and divided Attention**		**Factor 3: Executive functioning**		**Factor 4: Lexical and semantic abilities**	
**Test variable**	**Loading**	**Test variable**	**Loading**	**Test variable**	**Loading**	**Test variable**	**Loading**
Digit span forward	**0.740**	Ruff 2&7 (speed)	0.375	TMT-A (duration)	0.410	TRT	**0.808**
Digit span backward	**0.522**	Ruff 2&7 (accuracy)	0.351	TMT-B (duration)	0.202	Vocabulary test	**0.890**
Digit span sequential	**0.652**	TAP (auditive reaction time)	**0.537**	STROOP (reading interference)	**0.682**	LDT (reaction time difference)	**0.678**
2-back task (reaction time)	0.451	TAP (visual reaction time)	**0.710**	STROOP (naming interference)	**0.804**		
2-back task (errors)	**0.700**	TAP (errors)	0.401				
Reading span (% correct)	0.378	WAF-S (reaction time)	**0.720**				
		WAF-S (errors)	0.490				

*Factor loadings higher than 0.5 are highlighted with bold font*.

### Link between cognitive abilities and speech recognition

To predict performance in the speech recognition tests, stepwise linear regression analyses were performed based on the data of all 41 participants. Because the SRT data demonstrated that hearing loss had an influence on the participants' performance, PTA4 was always added first to the model, followed by the four cognitive composite factors (see Table 4). The order of entering the cognitive factors was driven by different additive sequences of pre-analyses, targeting toward a high *R*^2^ in the final models. PTA4 was predictive of the participants' performance in all five listening conditions, despite the fact that EHI listeners were aided. After *p*-value correction (Benjamini–Hochberg procedure with *Q* = 0.25, Benjamini and Hochberg, 1995), only one model included a significant contribution of cognitive factors in addition to PTA4. This was the model obtained for condition E (intelligible, single-speaker maskers in cafeteria noise), in which the lexical abilities factor was predictor for the SRTs once PTA4 was controlled for.

**Table 4:** Results of the stepwise regression analyses for the data of all participants (*N* = 41) calculated for each listening condition (A–E).

**SRT**	**Predictors**	**B**	***R*^2^**	**Adjusted *R*^2^**	***R*^2^ change**	**Significance**
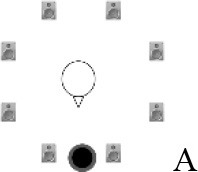	**PTA4**	**0.822**	**0.676**	**0.667**	**0.676**	**<0.001**
	MEM	0.104	0.684	0.667	0.008	0.280
	ATT	−0.022	0.684	0.658	0.000	0.836
	EX	0.046	0.685	0.649	0.001	0.801
	LEX	0.129	0.689	0.642	0.004	0.273
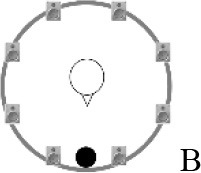	**PTA4**	**0.845**	**0.715**	**0.707**	**0.715**	**<0.001**
	MEM	0.046	0.716	0.701	0.001	0.613
	ATT	−0.022	0.722	0.699	0.006	0.390
	EX	0.038	0.722	0.691	0.000	0.824
	LEX	0.151	0.724	0.683	0.002	0.175
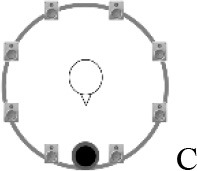	**PTA4**	**0.785**	**0.616**	**0.606**	**0.616**	**<0.001**
	MEM	0.141	0.635	0.615	0.019	0.173
	ATT	−0.095	0.643	0.613	0.008	0.396
	EX	0.156	0.649	0.609	0.006	0.422
	LEX	0.163	0.649	0.594	0.000	0.190
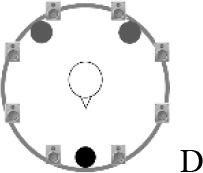	**PTA4**	**0.803**	**0.646**	**0.636**	**0.646**	**<0.001**
	MEM	0.050	0.652	0.633	0.006	0.619
	ATT	−0.085	0.658	0.629	0.006	0.433
	EX	0.166	0.665	0.627	0.007	0.379
	LEX	0.172	0.670	0.620	0.005	0.152
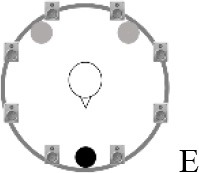	**PTA4**	**0.842**	**0.709**	**0.701**	**0.709**	**<0.001**
	MEM	0.078	0.714	0.698	0.005	0.392
	ATT	−0.102	0.722	0.699	0.008	0.302
	EX	0.369	0.740	0.722	0.018	0.040
	**LEX**	**0.277**	**0.767**	**0.732**	**0.027**	**0.008**

*Significant results are highlighted with bold font*.

### Effect of hearing loss and its interaction with cognition

To compare the effects of cognition on speech recognition in the presence or absence of hearing loss, linear regression analyses were also performed for the two groups separately (for 20 or 21 participants). Again, PTA4 was entered first in each model, followed by the four composite cognitive factors. The results are shown in Table 5 for the ENH group and in Table 6 for the EHI group.

**Table 5:** Results of the stepwise regression analyses for the data of the ENH group (*N* = 20) for each listening condition (A–E).

**ENH**						
**SRT**	**Predictors**	**B**	***R***^2^	**Adjusted** ***R***^2^	***R***^2^ **change**	**Significance**
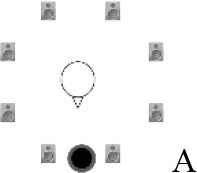	PTA4	0.348	0.050	−0.003	0.050	0.345
	MEM	0.071	0.101	−0.011	0.051	0.509
	ATT	−0.100	0.155	−0.014	0.054	0.344
	EX	0.217	0.202	−0.026	0.047	0.382
	LEX	0.005	0.202	−0.105	0.000	0.958
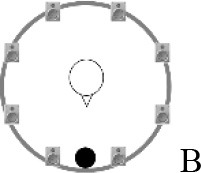	PTA4	0.330	0.035	−0.019	0.035	0.430
	MEM	−0.016	0.069	−0.047	0.034	0.899
	ATT	−0.206	0.250	0.100	0.181	0.076
	EX	0.411	0.382	0.206	0.132	0.105
	LEX	0.010	0.383	0.146	0.001	0.916
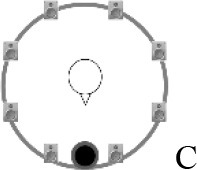	PTA4	0.268	0.017	−0.037	0.017	0.582
	MEM	−0.051	0.069	−0.048	0.057	0.714
	ATT	−0.195	0.195	0.034	0.126	0.147
	EX	0.411	0.297	0.096	0.102	0.176
	LEX	0.046	0.307	0.040	0.010	0.674
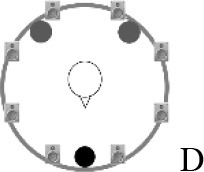	PTA4	0.379	0.046	−0.007	0.046	0.364
	MEM	−0.012	0.187	0.086	0.141	0.910
	**ATT**	−**0.244**	**0.476**	**0.371**	**0.289**	**0.012**
	EX	0.235	0.525	0.389	0.049	0.250
	LEX	0.051	0.542	0.367	0.017	0.491
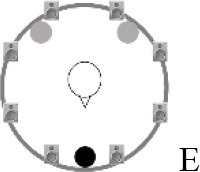	PTA4	0.657	0.101	0.056	0.101	0.103
	MEM	0.126	0.193	0.105	0.092	0.230
	ATT	−0.240	0.452	0.343	0.259	0.041
	EX	0.286	0.503	0.361	0.051	0.253
	**LEX**	**0.197**	**0.684**	**0.563**	**0.181**	**0.017**

*Significant results are highlighted with bold font*.

**Table 6:** Results of the stepwise regression analyses for the data of the EHI group (*N* = 21) calculated for each listening condition (A–E).

**EHI**						
**SRT**	**Predictors**	**B**	***R***^2^	**Adjusted** ***R***^2^	***R***^2^ **change**	**Significance**
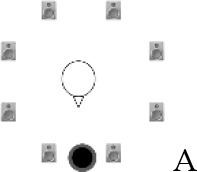	**PTA4**	**1.465**	**0.541**	**0.517**	**0.541**	**<0.001**
	MEM	0.176	0.582	0.535	0.041	0.201
	ATT	0.115	0.594	0.522	0.012	0.486
	EX	−0.099	0.598	0.498	0.004	0.689
	LEX	0.406	0.623	0.488	0.025	0.207
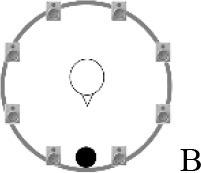	**PTA4**	**1.203**	**0.467**	**0.438**	**0.467**	**0.001**
	MEM	0.109	0.487	0.430	0.020	0.411
	ATT	0.033	0.488	0.398	0.001	0.841
	EX	−0.107	0.494	0.368	0.006	0.663
	LEX	0.519	0.571	0.417	0.077	0.102
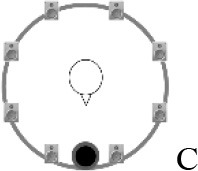	**PTA4**	**1.180**	**0.382**	**0.350**	**0.382**	**0.003**
	MEM	0.271	0.488	0.431	0.106	0.070
	ATT	0.026	0.489	0.399	0.001	0.882
	EX	0.028	0.489	0.361	0.000	0.915
	LEX	0.281	0.489	0.306	0.000	0.433
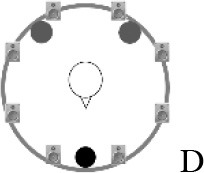	**PTA4**	**1.505**	**0.548**	**0.524**	**0.548**	**<0.001**
	MEM	0.129	0.569	0.521	0.021	0.360
	ATT	0.133	0.585	0.512	0.016	0.436
	EX	0.052	0.586	0.482	0.001	0.839
	LEX	0.343	0.603	0.461	0.017	0.313
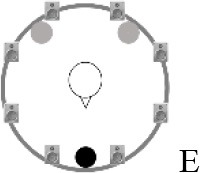	**PTA4**	**1.173**	**0.441**	**0.412**	**0.441**	**0.001**
	MEM	0.143	0.476	0.417	0.035	0.291
	ATT	0.045	0.478	0.386	0.002	0.784
	EX	0.064	0.480	0.350	0.002	0.798
	LEX	0.455	0.528	0.359	0.048	0.156

*Significant results are highlighted with bold font*.

For the ENH group, attentional skills were significantly predictive in a listening condition with spatially separated signals (condition D) after applying a correction for multiple testing (Benjamini and Hochberg, 1995). The influence of attentional skills was negative, meaning that better speech recognition was associated with poorer attentional abilities. Furthermore, good lexical and semantic abilities were positively correlated with better speech recognition in the cafeteria situation with informational masking (condition E). Neither PTA4 nor the other composite cognitive abilities were predictive of speech-in-noise performance. For the EHI group, variance in the SRTs is only explained by PTA4.

## Discussion

### Speech recognition

Differences in SRTs between the two groups were found, even though all EHI listeners were tested with bilateral hearing aids fitted according to NAL-NL2. This is consistent with the observation that supra-threshold auditory processing is associated with speech-in-noise perception, independent of aiding (e.g., Moore et al., 2012; King et al., 2014; Füllgrabe et al., 2015). As no real-ear insertion gains were measured in the current study, it cannot be ruled out that audibility did play a role. Concerning the use of hearing aids during the SRT measurements, some additional aspects could have led to perceptual differences between the groups. Due to the positioning of the hearing aid microphones above the outer ears, the EHI listeners might have benefitted less from pinna cues in the spatially complex conditions than the ENH group. Secondly, the amplitude compression in the hearing aids could have led to an impaired segregation of the speech signals (e.g., Stone et al., 2009) and smeared amplitude envelope cues (e.g., Souza, 2002). Therefore, the EHI group could, in principle, have benefitted less from dip listening due to the time constants of the hearing aid processing. As the release time of 90 ms is rather fast-acting, this effect should have been small. Nevertheless, since release times might be longer in real-ear listening (compared to the technical specifications in the data sheet), the pauses in the IFFM masker (with a maximum pause length of 250 ms) might have been too short to perceive the speech signal in the temporal gaps, especially in condition A. In conditions B-E, the masker signal was more continuous, which probably reduced the impact of the compression release time on speech-recognition outcomes. An additional consideration is that the EHI participants were not acclimatized to the given hearing aids. This could have led to poorer thresholds in the SRT measurements. However, the participants were acclimatized to general amplification and hearing-aid processing, because all EHI were experienced hearing-aid users. In addition, since hearing-aid functionality was limited to non-linear amplification, while deactivating other adaptive signal processing algorithms, any signal distortions and hence detrimental effects of the hearing aids were kept to a minimum. Nevertheless, the use of hearing aids is cognitively taxing (Lunner et al., 2009) and might therefore have a detrimental effect on SRTs in a complex listening condition. Another reason for the group differences in SRTs might be the outperformance of the ENH in most of the cognitive tests (see Table 2). Although the observed differences were not statistically significant, it cannot be ruled out that this group difference also led to a better performance of the ENH compared to the EHI in the SRT measurements.

Unexpectedly, no significant differences were observed among several listening conditions. The listening conditions were constructed to be sensitive to the effects of dip listening, spatial separation and informational masking. For dip listening (conditions A vs. C), a significant difference in SRTs was found but these conditions also differed in terms of their spatial properties and thus the difference might have been even larger if masking in condition C were not reduced due to the spatial percept. While the spatial separation of maskers (conditions C vs. D) led to SRT changes, no significant difference between listening to the IFFM (condition D) compared to a real conversation (condition E) was found. As described earlier, informational masking was operationally defined here as the introduction of semantic information. Another dimension of informational masking is related to auditory object segregation, which was represented in the difference between conditions B and C. Here, SRTs were higher (poorer) when auditory object segregation abilities played a role due to the presence of the IFFM masker. In the presence of the understandable conversation as an informational masker with additional semantic information but no differences in auditory object segregation a tendency to lower (better) SRTs was found compared to the incomprehensible IFFM masker condition. This finding is contrary to the expectation that the additional semantic information would lead to a more challenging condition and to a higher variance in SRTs (Durlach et al., 2003). One possible explanation for this observation could be that the IFFM was unfamiliar to the participants. After the measurements, some participants informally pointed out that they perceived this unfamiliar signal to be difficult to suppress when concentrating on the target signal. Furthermore, the IFFM is a highly fluctuating signal with many changes and onsets due to the different speakers in the signal. Therefore, it is very unpredictable, while the conversation that was used as informational masker in condition E was uniform and might be more easily suppressed by the participants. In addition to that, the higher rate of onsets of the IFFM compared to the conversation might have led to a disturbance in the suppression of the masker signal because of the repetitive directing of attention to the masker.

### Cognitive abilities and speech recognition in elderly listeners

The PTA4 of the 41 elderly participants had significant predictive power in the SRT models for all listening conditions. This is in line with the speech-recognition outcomes that showed higher (poorer) SRTs for the EHI group than for the ENH group. Additional significant predictive power of cognitive abilities was found only in condition E, in which the cafeteria noise and the realistic conversation were used as maskers. In this listening condition, lexical abilities slightly (but significantly) contributed to the model, with an *R*^2^ change of 2.7%. As this was the most complex listening condition, a stronger link to cognition was expected compared to the standard listening conditions used in speech audiometry. The magnitude of the *R*^2^ changes due to the inclusion of cognitive variables are quite similar to findings in the literature regarding elderly participants with mild sensorineural hearing loss examining their speech recognition of everyday-life sentences in modulated noise (Heinrich et al., 2015). Nevertheless, based on studies in which aided SRTs were measured (Humes et al., 2013; Heinrich et al., 2016), the effect of cognitive abilities on aided measurements was expected to be larger and that of PTA to be smaller than actually observed. This might indicate that audibility was not fully ensured (as mentioned above) and therefore masked the effects that were to be measured here. Another possibility that was not considered in the study is that supra-threshold auditory processing abilities might be substantially reduced in the EHI. For future work, the influence of these types of abilities should be included in the test battery to gain deeper insights into how such abilities contribute to speech recognition in noise.

In the recent research literature, inconsistent findings are reported concerning the link between working memory and speech recognition in noise. A literature overview of Besser et al. (2013) reported that reading span scores were associated with speech recognition in a number of studies with hearing-impaired participants or normal-hearing groups of a rather wide age range (e.g., 18–50 years in Ellis and Munro, 2013 and 40–70 in Koelewijn et al., 2012). In contrast, the meta-analysis of Füllgrabe and Rosen (2016b) showed that this does not necessarily hold for normal-hearing participants. One explanation for this difference might be that many studies investigating participants with a wide age range that were reported by Besser et al. (2013) did not control for age in their analysis, while Füllgrabe and Rosen (2016b) conducted the statistical analysis in narrower age groups or with partial correlations controlling for age. Furthermore, in the data surveyed by Besser et al. (2013; NH, age-range: 18–78) the significance of the link between reading span scores and SRT in fluctuating noise vanished when controlling for age. In the current study, no link between verbal working memory (e.g., reading span task) and understanding speech in noise was found, independent of the hearing status. This was neither the case in the regression models shown here, nor in bivariate or partial (controlled for age/age and PTA) correlational analyses of the data including reading-span scores with and without consideration of the item order (not reported here). These findings are incongruent to the expectations drawn from the ELU-model (Rönnberg et al., 2010). Comparing different normal-hearing persons having a wide age range, the correlation between working memory capacity (as measured with the reading span test) and speech recognition in noise was not significant in younger participants (18–39 years) but it was in the older age groups and it increased at higher age (Füllgrabe and Rosen, 2016a). A possible explanation for the low correlation between span scores and SRTs might be the relatively young “older” age of the participants tested here. The strongest correlation reported by Füllgrabe and Rosen (2016b) was found for the oldest group (70–91 years), which was the upper boundary of the age range considered in the present study. Nevertheless, Füllgrabe and Rosen (2016b) found some evidence for a link between working memory and speech-in-noise tests for comparable age groups as reported here which could not be replicated.

Only a few studies included more realistic free-field spatial listening conditions, such as used here. Keidser et al. (2015) administered an auditory test to measure the cognitive spare capacity and found an association with working memory in realistic cafeteria noise when controlling for PTA and with audio-visual stimuli, but not for spatially separated babble noise. Those findings were obtained in participants aged 22–49 and 67–77 using a more complex task than the speech recognition task in the current study. Due to this, it is not clear whether the differences in the findings are based on the masker difficulty or the task itself.

It could further be criticized that the neuropsychological test battery used here was not sufficiently differentiated or specialized to predict speech recognition in complex listening conditions. The tests used in this study were carefully chosen and based on recent literature. Although the cognitive tests were mostly created for diagnostic issues and therefore might not be suitable for scientific purposes, no ceiling effects were observed in this study. However, it cannot be ruled out that the use of diagnostic testing procedures might have led to the unexpectedly weak links between the cognitive factors and SRTs in most listening conditions. To avoid effects of too general testing procedures, a better approach for future research might be an experimental design to investigate the link between speech recognition and cognition instead of correlational analyses.

### Elderly listeners with and without hearing loss

The results of the regression analyses calculated separately for the two participant groups differed from the findings described in the previous section. For the ENH participants, the cognitive factor ATT was predictive in condition D with IFFM from ±135° and contributed to the model with an *R*^2^ change of about 29%. Modeling the SRTs in condition E with the intelligible dialogue as spatially separated maskers, only LEX (*R*^2^ change: 18.1%) had predictive power. PTA4 was not predictive for any SRT outcome of this group, which can be explained by the low variance in pure-tone thresholds (see Figure 1). It is in line with the findings for the whole-group regression model that lexical abilities play a role in speech recognition with informational masking. Participants with greater vocabulary and faster lexical access benefited from listening to understandable maskers compared to the IFFM masker and to their peers that had lower lexical abilities.

Surprisingly, the factor ATT contributed negatively to the model for condition D. This means that participants with better attention-test outcomes (mostly divided attention, see factor solution in Table 2) were poorer at recognizing speech under this particular spatially separated masker condition. Overall, the relatively small sample size in this study has to be considered when interpreting these results, as well as the repeated regression models for the two subgroups. Although significance levels were controlled for repeated analyses, the group size might have been too small to calculate reliable regression models. In addition, the sampling of the participant groups might be biased, which could have led to the unexpected results. Nevertheless, the age-matched groups were carefully recruited and cognitive testing as well as listening conditions, were systematically chosen based on literature findings. The findings regarding between-group differences in SRTs as well the threshold differences due to different complex listening conditions show the need for a theoretical approach regarding the relevance of cognitive abilities in daily-life listening conditions. A possible theoretical explanation of the observed effect is that faster processing speed in switching between the two modalities (in the divided attention test) and higher automatic processing speed (in the selective attention task) might be associated with higher sensitivity to interference in certain tasks (Ansorge and Leder, 2011). Concerning this special ability, the continuously occurring signal onsets in condition D might have distracted the participants with good attentional skills more than the participants who were not able to cope with the dynamic changes in the signal.

Although the EHI participants were aided during all auditory measurements, PTA4 was the predictor with the highest power and *R*^2^ changes from 38.2 to 54.8%. This reproduces earlier findings (Heinrich et al., 2016). No additional variables contributed to any of the listening conditions. One possible explanation for this might be that hearing loss and cognitive abilities are related, which was reflected in the mostly poorer cognitive performance of the EHI compared to the ENH participants (see Table 2). Although the differences were not statistically significant because of the high variance, it cannot be ruled out that by controlling for PTA4 in the regression models, effects of cognition are also covered by the factor PTA4. The expectation arising from the ease of language understanding model (ELU) that degraded signals lead to higher cognitive load (Rönnberg et al., 2010) was not fulfilled in this study. Rather than a stronger link between cognition and speech recognition for the EHI group, hearing loss appeared to explain most of the variance in the data, thereby masking the putative effects of cognition in statistical analyses. This replicates earlier findings (Smith and Pichora-Fuller, 2015), but is also inconsistent with others in which an influence of cognition (especially working memory) was found, although a similar sample of aided hearing-impaired participants was tested (Lunner, 2003). Humes et al. (2013) found clear effects of cognition on speech recognition with spectrally shaped speech material (i.e., well-controlled linear amplification up to 8,000 Hz). It is therefore possible that the amplification provided by the hearing aids used here was insufficient to reduce the influence of audibility on speech recognition. Beyond that, although participants were familiar with amplification, they were not acclimatized to the fitting used during the speech recognition measurements. This could have obscured the results. Further research is needed to resolve this issue.

## Conclusion

This study explored the question of which specific cognitive abilities are linked to the speech recognition of elderly persons in listening situations more complex and ecological than those commonly used in laboratory studies. Contrary to our expectations, relationships of attentional and linguistic abilities were only found for the ENH group when using speech-in-noise tasks with spatial separation, while for the EHI group no link between speech recognition and cognition was found. Furthermore, no significant link between speech recognition and working and short-term memory was found. Overall, this implies that the involvement of cognitive functions in speech recognition in complex listening conditions is still unclear. The results also indicated that if the masker contains at least partly intelligible speech, lexical abilities for speech recognition may be helpful. Finally, ENH participants with better attentional abilities obtained poorer speech recognition outcomes under a spatially separated condition, requiring further research to better understand this and the abovementioned effects.

## Ethics statement

The study and protocol were reviewed and approved by the Kommission für Forschungsfolgenabschätzung und Ethik of the Carl von Ossietzky University in Oldenburg, Germany (Drs. 22/2014). Written informed consent was obtained from all participants in accordance with the Declaration of Helsinki.

## Author contributions

ThN, ToN, and IH: designed the study; ThN: conducted the measurements, analyzed the data and wrote the manuscript; ToN, IH, and RS: contributed to critical discussions and revised the manuscript.

### Conflict of interest statement

The authors declare that the research was conducted in the absence of any commercial or financial relationships that could be construed as a potential conflict of interest.
